# Annotations

**Published:** 1889-08-24

**Authors:** 


### Annotations.

**What should the Deaf do?** We published last week a letter under this heading from Mr. Morris Jones. Among other things, Mr Jones said : "There are thousands who highly value the useful information given in The Hospital on different departments of medicine, surgery—epilepsy, eyes, etc. Some account of what can be done in the case of deafness and dumbness would be very acceptable. . . . . There are large numbers of children deaf, or partly, and the parents know not what to do. . . . . Anything said that will enable parents to distinguish between hopeless cases and those that could possibly benefit by going to London or some town where there are specialists would be very valuable." A single word might almost answer our correspondent's difficulties. Every deaf and dumb child should be taken to a competent specialist at the earliest possible moment. Mr. Morris Jones thinks the ordinary general practitioner a little backward in his knowledge of ear cases. The ordinary general practitioner, however, very seldom professes special knowledge and skill in deafness or dumbness, and is generally the first to suggest a visit to a specialist. In London every general hospital has a special department for ear and throat diseases, and a competent surgeon in attendance. In all the principal provincial hospitals the same rule obtains. So that there is no reason why the poorest parent should not soon find out for himself whether his child is temporarily or hopelessly deaf. Every parent ought to know that what is called "throat deafness' is generally curable if promptly and vigorously treated; whilst if left to itself it may, with repeated colds, end in permanent inability to hear. If, for example, a child has large tonsils and becomes deaf whenever he has a cold, or when the weather is damp and chilly, or when his health is feeble, he is probably a sufferer from "throat deafness." It is impossible to explain to those who have no knowledge of anatomy the nature of the organic disease or defect or excess of structure which may produce permanent deafness. It is, however, a safe rule, and one that should never be infringed, to let every deaf and dumb child in every rank of life have the advantage of a thorough examination by a competent specialist. He who is responsible for a deaf child, and does not secure for him this advantage, at least once, may rightly be judged to have done the child a grievous and possibly an irreparable injury.

**Troubled Waters.** The council of the British Medical Association had a difficult hill to climb at the annual meeting of the association held at Leeds last week. They climbed it, however, very carefully, and reached the top with distinct success. The *British Medical Journal* early in the year, when all the world was excited by the quarrel which went on week after week between English and German doctors, published a famous "script." Certain members of the profession in London, some of them of excellent character and judgment, and a few of considerable eminence, were gravely displeased by the publication of the script, and thought that it showed an unjustifiable lack of courtesy towards the medical profession of Germany. That feeling was right; and the conductors of the *British Medical Journal* acknowledged it to be so; and, moreover, they courteously apologised in the proper quarter. There the incident should have ended. But some of the dissatisfied ones, being more censorious than wise, and apparently feeling that there had by some means been committed to their keeping the special duty of taking care of the honour and dignity of the medical profession, approached the duly-elected council of the British Medical Association in much the same manner as a superior village schoolmaster might have approached a batch of unkempt wearers of corduroy who had added to the enormity of playing truant the sin of stealing ripe gooseberries. The good sense of the members of the British Medical Association at once asserted itself. They perceived that, however much the dissatisfied few might have been justified in their original complaint, they had now exceeded the bounds not only of good manners but of common sense. The malcontents were willing to prove their desire to make atonement to half-a-dozen members of the profession in Germany by convicting half a hundred members of the profession in their own country of journalistic incompetency and want of professional dignity. The spectacle is instructive. It is no secret that some of the malcontents are much readier to take counsel of their own self-esteem than of plain sense, and of a due and philosophic regard for the feelings of others. Professional dignity is a much-worshipped fetish in certain quarters; and, like other and less worthy superstitions, it is very apt to claim for itself the surrender both of common-sense and brotherly feeling. The British Medical Association, with only one dissentient, to put it plainly, "snubbed" the malcontents. It is for the latter now to consider whether or not they did not distinctly invite such a "snub." In our judgment they certainly did; and if they feel as much regard for their own true personal dignity as they profess to feel, and probably do feel, for the honour of the medical profession, they will lose no time in offering to the council of the British Medical Association such an apology as justice and brotherly feeling demand.

**A Court of Criminal Appeal.** If a man lose a lawsuit which involves £50, he may by the constitution of this country appeal to a higher court; but if he be tried for his life and condemned by a jury of twelve of his fellow-countrymen, he has no appeal to any court whatever. We are not among those who affect to look down with lofty contempt from the height of their own superiority on the plain British citizen in his capacity of juryman. Trial by jury is an institution older than Magna Charta, and that which has weathered the storms of civil wars, changes of dynasty, constitutional reforms, and social developments, and has kept its place unmoved in all those varying circumstances, may very well afford to smile at the vapouring criticisms of beardless lawyers or the superior scorns of medical and scientific experts. A trial by a jury of his peers and equals affords to the average man when accused such security for a fair hearing and an impartial verdict as he will not readily forego. But whilst such a trial is what the ordinary citizen will most trust and prefer, it does not necessarily follow that the verdict of a jury of one's peers is always infallibly correct. On the contrary, it has been proved in not a few instances that innocent men have had to undergo the last penalty of the law whilst guilty ones have escaped. The Maybrick case is regarded by many as an instance of a complete failure of justice, both on the part of the judge and the jury. We ourselves do not so regard it. On the contrary, it seems to us that the summing up of the judge was in strict accordance with fairness, and the decision of the jury equally in accordance with facts. But if the Maybrick case concentrates attention on certain anomalies in our legal system, and results in their removal, trial by public opinion will have served at least one good purpose. It is not necessary to do more than affirm that if an adverse decision in a case where property is concerned may be questioned, and the case carried to a higher court, much more should it be possible for a cause to be re-heard when life itself is at stake. In order to make this convincingly clear to himself, a man has only to imagine that he has been wrongfully charged with murder, and that circumstantial evidence having gone against him, he is now condemned to be hanged. There is another anomaly equally flagrant, though not always so fraught with serious consequences; and that is the hearing and deciding of cases involving special sciences—such, for example, as medical cases by judges—who have no knowledge of such sciences. This often involves the grossest injustice. A medical case was tried not long ago in which the counsel for the winning side declared afterwards that he was convinced his side was in the wrong, and that they had never expected to get a verdict. The medical evidence was such that any competent medical man would have decided against the winners even on the evidence of their own witnesses. A lawyer is no more competent to try a medical case legally than he is to treat it professionally. To ask him to do so is just as foolish as it would be to ask him to prescribe for typhoid fever or to perform ovariotomy. If lawyers deserved the name of jurists they would be the first to see this. In any medical case of importance, and indeed, in every such case, the ordinary judge should always have at his side a competent medical assessor. In no other way is it possible for justice to be done.

